# Organisational structures and processes for health and well-being: insights from work integration social enterprise

**DOI:** 10.1186/s12889-022-13920-4

**Published:** 2022-08-27

**Authors:** Andrew Joyce, Batool Moussa, Aurora Elmes, Perri Campbell, Roksolana Suchowerska, Fiona Buick, Jo Barraket, Gemma Carey

**Affiliations:** 1 Centre for Social Impact, Swinburne University of Technology, Mail H25, Cnr John and Wakefield Streets, PO Box 218, Hawthorn, VIC 3122 Australia; 2 School of Business, University of New South Wales, Northcott Drive, Canberra, ACT 2600 Australia; 3 Melbourne Social Equity Institute, University of Melbourne, Grattan Street, Parkville, VIC 3010 Australia; 4 Centre for Social Impact, University of New South Wales, UNSW Sydney, 704, Level 7, Science Engineering Building, Sydney, NSW 2052 Australia

**Keywords:** Young people, Economic and social inclusion, Workplace health and well-being

## Abstract

**Background:**

Previous research on employee well-being for those who have experienced social and economic disadvantage and those with previous or existing mental health conditions has focused mainly on programmatic interventions. The purpose of this research was to examine how organisational structures and processes (such as policies and culture) influence well-being of employees from these types of backgrounds.

**Methods:**

A case study ethnographic approach which included in-depth qualitative analysis of 93 semi-structured interviews of employees, staff, and managers, together with participant observation of four social enterprises employing young people.

**Results:**

The data revealed that young people were provided a combination of training, varied work tasks, psychosocial support, and encouragement to cultivate relationships among peers and management staff. This was enabled through the following elements: structure and space; funding, finance and industry orientation; organisational culture; policy and process; and fostering local service networks**.**. The findings further illustrate how organisational structures at these workplaces promoted an inclusive workplace environment in which participants self-reported a decrease in anxiety and depression, increased self-esteem, increased self-confidence and increased physical activity.

**Conclusions:**

Replicating these types of organisational structures, processes, and culture requires consideration of complex systems perspectives on implementation fidelity which has implications for policy, practice and future research.

## Introduction

Employment is considered one of the key determinants of health and well-being [1] and relates to other influential social conditions such as education, income, social status and material circumstances. Unemployment is associated with poverty, social isolation and worsened mental health outcomes [2, 3] and exclusion from decent employment limits social participation and opportunities for skill development [4], which has multiple negative effects on the economic and health status of individuals and communities [5]. One potential avenue for inclusive employment opportunities is social enterprise (SE) – or businesses that trade to fulfil a social mission [6]. Work integration social enterprises (or WISEs) have a primary social purpose of creating meaningful employment opportunities or pathways to employment for people who are disadvantaged in the open labour market [7], particularly for people with disabilities. SE scholars have theorized that WISEs may provide a pathway to address the social and economic inequities that contribute to illness, through mechanisms such as creating employment, and increasing peoples’ access to economic and social resources [7–9].

A current gap in this literature is an understanding of the specific organizational processes, structures, and culture of the workplace environment that either support or hinder health and well-being [10]. Some scholars have argued that workplaces that are inclusive – that is, those that enable all employees to feel a sense of belonging while still being confident to express individual identity related to ethnicity, gender, sexuality and other domains – support health and well-being [11]. However, there is a lack of empirical research into whether young people from diverse backgrounds and those with diagnosed mental health conditions are able to feel a sense of connection and belonging in the workplace and what impact this has on their health and well-being. Even social enterprises, which are explicitly concerned with promoting inclusivity and social benefit, are only just starting to receive attention from researchers on how they promote health and wellbeing among stakeholders [10].

The aim of this research was to address these gaps in the literature by analyzing the organisational strategies that WISEs utilise to support the health and well-being of young people that have previously been excluded from the labour market. The focus on young people was due to their higher rates of unemployment and underemployment relative to general population [12], and where there is an opportunity to address risk factors which can have a positive impact on current and future mental health [13]. The main research question explored in this paper was: What are the organizational structures, processes, and culture that enable WISE to employ young people who have experienced economic and social disadvantage and how do these organisational elements impact on the health and well-being of these young people? This paper was part of a larger study examining how social enterprises redress social determinants of health inequities among young people.

The paper will outline previous research on the health benefits of employment and where there are gaps in relation to understanding *how* particular organisational strategies either promote or hinder positive well-being among employees. Through in-depth qualitative analysis of 93 semi-structured interviews and field note observations, the findings show how employees perceived a number of positive changes to their mental and physical health which they attributed to certain organisational strategies related to processes, structures, and culture. The paper will also present challenges for future research and practice on how to further develop and test the findings presented in this paper.

## Background

### Employment as a Social Determinant of Health

While employment is considered a social determinant of health [14], there are mixed findings on whether employment itself has a positive effect on mental health [15]. This relationship between employment and mental health varies according to a number of factors such as job security, the quality of the work, the level of control of the work tasks, and whether it is meeting the individual’s personal needs [16, 17]. Current social determinants of health models do not address this level of complexity and often present employment itself as a positive contributor to well-being wherein the reality is more nuanced [14, 18]. The quality and nature of the employment is particularly important for young people (typically, classified as 15–24 years) as risk and protective factors for mental health at this point in someone’s life can have substantial impact on future health and well-being [19].

While the research is still emerging, there is some work to suggest that social enterprises are able to provide employment and training opportunities for people previously excluded from the labour market and that there is some benefit for their mental health and social capital [20, 21]. There has been little research examining the impacts for young people although some studies have found that social enterprise interventions can have a positive effect on the mental health of young people [22, 23]. What is currently lacking from this research is the specific organisational factors influencing these health gains [10, 24, 25] and the voices of young people themselves with the perspectives of social enterprise managers and funders currently dominating the research base [26, 27]. One of the proposed mechanisms for *how* social enterprises enable positive mental health of employees is through providing an inclusive workplace environment [7–9].

### Organisational Structures, and Health and Well-Being of Employees

Work integration social enterprises provide a useful organisational type to explore how organisations can, through the design of organisational structure, processes and culture, promote the health and wellbeing of people from disadvantaged backgrounds [7]. Social enterprises are organisations where one of the main goals is promoting social or environmental benefit while ensuring the business is profitable [28]. A systematic review conducted by Roy et al. [9] found some (albeit limited) evidence from Australia, Canada, Hong Kong and the USA of social enterprise activity positively impacting on health and well-being. Specifically, involvement in social enterprise improved people’s mental health, self-reliance/esteem and health behaviours, reduced stigmatization and built social capital. Scholars are starting to explore the organisational features of social enterprise that enable them to achieve these health and wellbeing outcomes [10, 29, 30].

Suchowerska et al. [10] theorise that organisations impact health equity and well-being through two distinct processes. Transformational processes, which are shaped by organisations’ leadership, culture and mission, put pressure on social structures and institutions that entrench health inequities. Transactional processes, which are shaped by the relational, structural and policy mechanisms of an organisation, can more rapidly shift the quality of life, wellbeing and self-efficacy of individuals within the organisation. This whole-of-organisation perspective contrasts with prior research that has tended to focus on how specific programs within organisations impact workplace inclusion and in turn, health equity [10].

The aims of this research were to examine in further depth how the structure, operation, and culture of an organization itself influences health and well-being outcomes. In doing so the intention is to illuminate the core features of good WISE practice that can explain how a WISE achieves social and health impact [31] and to offer suggestions for future workplace well-being practice and policy based on these findings. As both a topic that has received little research focus from an organizational perspective and a participant group that is more likely to feel disenfranchised, qualitative research was deemed important for giving ‘voice’ to this group and exploring organizational processes and strategies in more depth [32–34]. The research question that guided this study was: What are the organizational structures, processes, and culture that enable WISE to employ young people who have experienced economic and social disadvantage and how do they impact on their health and well-being?

## Methods

The data presented in this paper is from a three-year research project funded by the Australian Research Council through its Linkage Scheme. The project focused on the health and well-being impacts of Australian WISE on young people aged 15 to 24 who have experienced some form of disadvantage related to education and employment opportunities [13, 35]. This age group experiences higher rates of unemployment and lower rates of participation in the employment market relative to general population averages [36].

### Case study research design

This study required exploration of specific features of workplace design and structure and how these features were experienced by young people and the perceived impact on their health and well-being. In order to understand and explore this particular type of workplace structure, it was important to examine it in situ and understand critical contextual factors, social processes and dynamics [37, 38]. Thus, a case study approach was important in order to facilitate understanding and to provide a boundary around the subject of investigation [37].

Case studies were selected based on a paradigmatic case sampling approach [39], which seeks to include examples that demonstrate prototypical characteristics of the phenomena in question. The paradigm being explored is the interaction between SE operations and employment experience and health outcomes for young people [14]. The four WISEs selected were located in the Australian states of New South Wales (NSW) or Victoria and operated within or into areas experiencing locational disadvantage, as defined by the Australian Bureau of Statistics [40] SEIFA index. These States were selected because they have the highest concentration of SEs in Australia [6]. Each of the WISEs had been in operation longer than five years and were well-established in respect of organizational culture, structure and processes. The location and industry of each case study were:

- Case A: Inner-Metropolitan Melbourne, Hospitality
- Case B: Inner-South Sydney, Information technology and electronics
- Case C: Greater Melbourne, Construction
- Case D: South Coast New South Wales, Farming and Waste management

Young people participating in training or working at the WISE had diverse backgrounds. Three of the organisations had successfully engaged refugees and immigrants in their programs, and all organisations engaged young people with mental health issues. One of the WISEs recruited participants directly from local schools, while the others also included young people who had exited school. Given that the research problem being examined requires rich analysis of organizational factors and their effects, ethnographic data collection methods were used. Ethnographic research enables researchers to engage with participants in their natural environments and, in line with a realist approach, understand what works for whom under what conditions. This approach can develop rich insights through ‘thick description’ [41] and help reveal both intended and unintended effects of practice [42]. This is consistent with both public health and institutional scholars’ calls for understanding organizational effects at the ‘coalface’ of practice [42, 43] and for more qualitative research to explore in-depth how organizational processes and dynamics are experienced by employees [32].

Thus a range of methods consistent with an ethnographic approach were undertaken, including: initial workshops with staff and directors on their perception of organizational processes and outcomes; 93 semi-structured interviews with young people, WISE managers, WISE funding and external organisations which were the key component of the data collection [44]; up to three weeks of participant observation within each WISE; collation of organizational documents; and concluding engagement workshops to share and make sense of the findings. To ensure qualitative research rigor, each of the steps in the process of sampling, data collection processes, and sequencing of analysis, are explained according to best practice guidelines and recommendations [33]. All participants provided informed consent and the study was approved by the Human Research Ethics committee of Swinburne University of Technology.

### Data collection

#### Preliminary workshops

The research team facilitated a 90-min Theory of Change workshop with staff and managers at each social enterprise. The purpose of the workshops was: (a) to identify how case study organizations delivered social impact and value by reviewing their organizational Theory of Change; and (b) to revise the organizations’ Theory of Change to guide measurement of social impact, test assumptions and support strategic planning activities. These workshops provided WISE staff and managers (young people did not participate in these workshops) with the opportunity to reflect on their understanding of organisational aims and goals, and also helped researchers to refine research questions to the specific case study. The workshops were recorded and minutes taken. They helped to shape the specific interview schedules for participant groups and shaped the field note observations but they were not included as part of the data that was coded.

#### Semi-structured interviews

Ninety-three semi-structured interviews were undertaken with participants to understand if, and how, the WISE workplace environment supported their health and well-being. Semi-structured interviews were used to ensure consistency across interviews and adherence to areas of interest while allowing sufficient flexibility for the participant to respond [45]. The questions for young people included overall experience, what skills they developed, what they thought of the different roles, what they thought of the support, whether they noticed any benefits to their health and well-being or any negative outcomes, and how they experienced the social environment of the workplace. The interview questions for staff and other stakeholders were similar, although they were asked to reflect on their perceptions of the benefits and challenges for young people, the extent to which organizational structures and processes supported these young people, and areas requiring organizational change and improvement. Interviews were audio recorded.

All members of each case study organization – young people who received services, managers and employees of the WISE – were invited to participate in the study via a group email sent by internal contacts. Thus, a convenience sample was used, as participants were those who volunteered to take part in the study. Additional participants were identified using a snowball sampling technique where, at the end of each interview, participants were asked to recommend other potential participants [46]. Overall, the sample comprised 27 young people, 12 managers, 7 partners, 19 staff, 15 representatives from external organizations and funders, 7 board members, and 6 executive staff.

#### Participant observation

Another key data collection strategy was participant observation within four case study organizations, which lasted an average of 13 business days for each case study organization. Due to the nature of on-site activities, researchers were limited to only 3.5 days of participant observation in one of the case studies. The researchers observed a range of activities, including training/ work programs and board meetings, and recorded notes of their experiences. For each organization, the same researcher was assigned for all of the observation period. Detailed field notes were written at the conclusion of each day in the form of a diary record focusing on organisational structures and processes that were engaging young people (or not engaging as the case may be) and any observations on the social relationships between young people and between young people and staff and managers (that is both bonding and bridging social capital) [44, 47]. The field notes focused on: the roles of staff, the use of space, the activities undertaken and experiences of participants, and the atmosphere of the WISE. The notes provided a record of: key staff members roles, the spatial layout of the WISE, the ways in which staff and participants interacted with the spaces and when, the use of spaces and objects for training/work/other purposes, photographs of the WISE (rooms used, training tools), researcher interactions with staff members and participants, key events of the day as described by staff and participants, staff and participants responses to training and work throughout the day, researcher reflections on the atmosphere of the WISE and cultural norms of the WISE.

### Data analysis

Interview and field note data was coded in NVivo 11 using open, axial and selective coding [48]. All the data sources were included in this coding process inclusive of interview data, workshop data, and field notes. To increase confidence that the findings accurately reflected the views of participants, triangulation approaches were used: methods and data source triangulation (using more than one method and data source); and researcher triangulation (two or more researchers involved in coding) [49].

Authors PC and RS undertook the process of an inductive open coding which involved the following steps: reading through the data line-by-line and segregating into parts; looking for areas of similarity and difference between the parts of the data; and creating thematic groups based on the data [50]. One of the researchers had been involved in field note observations and the other researcher had not been involved in observation, this helped to balance intimate knowledge of the context and some research distance [44, 47]. These themes were then discussed as a research team and agreement reached on the preliminary set of themes. The next step was to conduct axial coding where different thematic segments were clustered together by authors PC and RS and broader themes related to the research questions were developed. This corresponds to a second order level of analysis from Gioia et al.’s [44] methodology approach the aim of which was to explore the organizational structures and processes that were in operation.

These themes were then tested through a number of supplementary checks to strengthen the credibility and integrity of the findings [33]. This involved a second round of 90 min workshops with staff and managers of each of the participating WISEs where the emergent findings were presented, and themes discussed. The purpose of these workshops was to provide organizations with insight into early findings and seek feedback about how to direct future analysis. A series of case study reports for each organization were produced as part of this process and a range of graphic presentations to illustrate the findings which were discussed with WISE members.

Lastly, a selective coding process [48], took place with authors AJ and PC coding the data on how the themes/concepts related to organizational strategies (developed in state 2) were related to perceived health and well-being outcomes. Concepts related to how organizational features might impact on health and well-being outcomes described in a previous theoretical paper guided this analysis [10]. Following an abductive research approach [51], the analysis focused on the perceptions of participants in how organisational processes and structures were influencing health and well-being outcomes.

## Results

The findings are structured according to the research question of the organizational features that enabled WISE to impact on health and well-being. The data analysis uncovered the following organizational features as being important in influencing health and well-being: *structure and space; funding, finance and industry orientation; organisational culture; policy and process; and fostering local service networks.*

### Structure and space

There were a range of organisational structures through which psychosocial support and skill development occurred. The youth programs team in one of the cases provided an organisational structure for psychosocial support. In other cases where a team itself was not in place, this support was provided differently through policies and processes which will be covered later.

Participants across all of the case organizations reported a deliberate strategy of extending the skills of the young people and having them confront new situations – including developing new work skills, periodically changing work teams and venues, and engaging in diverse customer-facing roles – to increase their self-confidence. Being able to provide a range of different roles at different sites was considered important for their skill development and self-esteem. Young people and staff felt respected and valued within the workplace and training environment:

*… I was very scared, so when I start with [WISE] they were very supportive, they were very helpful, so I feel secure, I feel like – how to say sometime when I need support … especially for something work here at first I didn’t know much how to do so if I did something wrong so they … explain to me clearly. So they show me not just explain to me, they show me how to do so that’s how I started to feel confident and so I start to improve other – like I know how to do other things and also after that when I apply for a job… so that’s how I start to build my confidence. (Case D, Young person 10)*

*I think the biggest thing is when we finish the first containers and I’m standing there, ‘We can do it actually! We have done all of this!’ So I was proud. I can do it! So it gives me confidence. (Case C, Young Person 4)*

The constant recognition and praise for developing skills was seen as critical for the development of self-confidence and self-esteem.

The spatial design of the case WISEs impacted positively on young people’s sense of well-being. Each of the WISEs used space differently to cater for the different mental health needs of the young people. There was one case study that had a significant amount of green space which was noted as beneficial for well-being:

*When I’m at home the environment is a lot different. It’s a lot more stressful, a lot more work. Everything’s “Go, go, go, go, go.” When I come here for volunteer work it was come here, chill, do work. It’s quiet. You hear birds. You’re always surrounded by nature sort of thing, so it’s just awesome. (Case D, Young Person 6)*

All case organizations included a number of hidden areas and lesser-used rooms which can help to reduce stress levels by providing a place for quiet and solitude when needed. Designated areas, like break rooms or games rooms created a more informal space for young people to interact. There was a sense that socializing was a key element of the work and education environment and this was actively encouraged as a means to build self confidence in young people. The sense of belonging and having a community to connect with was seen as beneficial for autistic people or those with previous experience of social isolation; and also, for people experiencing depression, anxiety, and general loneliness. These quotes reflected a common sentiment across the different organizations and young people who were interviewed:

*Something tragic happened back in 2013 and that kind of like I was going through depression and stuff over it, so that set me back a lot with career things … I went into a bad depression … some places I’ve worked I’ve had like the best boss ever, but then some places I've had like people I just don’t want to work for and help out. But here is like, it's definitely up there. I haven’t met a single person here that I've not liked or gotten along with yet. Everyone is great and nice. They’ll answer any question you have. They won’t make you feel bad for asking questions. Just really supportive and motivated to help you and learn. (Case B, Young person 9)*

*I suffer with severe anxiety, and I do get a little bit of deep depression. But since being here, that’s gone. I think it’s amazing. I’ve come here, and I’ve just got this role now where I want to be at work, I’m happy to be at work… I feel supported here. I can come here and I can have my little chats to people. (Case D, Young person 1)*

Feeling that sense of connection with other people was one of the key factors that people felt was responsible for improving their mental health and reducing feelings of anxiety and depression.

### Finance, Funding, and Industry Orientation

Providing these work and training opportunities within a flexible environment was made possible through a mix of revenue streams. This included commercial product offerings, internal investment through their parent organisation and/or grant funding through philanthropic and/or government partners. This was seen as constant challenge in operating this type of organizational model:

*‘Access to finance is my ongoing challenge always. The challenges of trying to scale these things and getting access to the right type of capital… the market’s just too embryonic to have the things in place that you need to be able to access the capital at the right time’. (Leadership, Case A)*

The sustainability of the organizations depended in large part on aligning within an industry supportive of this business type and being commercially competitive. One of the key focuses was aligning the social goals of the WISE with the chosen industry to ensure that there were employment opportunities in that industry in that region for young people. There were some concerns by staff though that the culture and gender norms of the industry in which two of the case studies operated may not be suitable for the young people involved in the WISE.

*Interviewer: people write about hospitality as quite a male dominated industry. And this isn't specific for social enterprise but more hospitality in general. What's your take on that?*

*Participants: … I think this is definitely one of the main workplaces where I see a little bit more equal in gender but everywhere else I've worked is I would say 80% male dominated for sure. (Case A, Young Person 11)*

It was also noted in one of the case studies that industry norms around smoking was a point of connection between young people and staff which was a concern from a health perspective. As one staff member told us:

*… we don’t have an area that separates the students from the staff. So, we all smoke but the thing is, we’ve only got one smoking area so it means that you’re out there having a smoke and all the students are out there having a smoke. (Case D, Program Staff 2)*

In addition to the negative impact of smoking, accessible healthy food choices were a challenge in some industries due the location of the workplace. In some industrial settings there were no healthy food options available.

A point of consistency across the case studies was an acknowledgment of diversity was important to the WISEs which maybe atypical of industry norms:

*It [Organization] is like a community, because at the first time when we started, the classmate that we had is all from different ethnic groups, like from different communities, different people, like the people who came from - they kicked out of school or they were on drugs and stuff, they’re disabled or something like that. You’re getting involved in a lot sort of people, you know, different sort of people. (Case C, Young Person 8)*

There was strong recognition of diversity and as detailed thematically, a strong desire to validate people’s differences. From field notes recorded this was observed through strong visual cues – including posters, staff profiles of visible diversity around the WISEs; use of iconic symbolism – such as pride colours – in workplace design; and purposeful integration of visual, textual and auditory organisational health and safety materials to support participants of all abilities and linguistic diversity. While this was undoubtedly seen as valuable from the perspective of both the staff and young people, a consistent concern was that it created an unrealistic expectation of the realities of ‘normal’ workplaces:

*We provide an environment here that is really rare in that people are just supported no matter what their identity is, what their background history. It’s a very supportive environment which in turn has its own unintended consequences down the track when it comes to putting them back out in the real world. (Case A, Leadership)*

This highlights the need for broader workplace reform and change to ensure that workplace inclusion becomes more common.

### Organisational culture

Organisational culture refers to the shared beliefs and values that influences the relationship interactions and practices within an organisation. It was made abundantly clear in the interviews that this sense of feeling comfortable and being able to be yourself was highly important to the staff and young people in the case organizations. There was a strong focus on people feeling safe to disclose any mental health conditions. In terms of authenticity, people felt comfortable being open about their mental health challenges and felt supported in doing so.

*Yeah, and be safe, feel safe and supported and nurtured and know if there’s baggage and many times there are, that can be left at the gate and just come in and have that free open mind and not be judged or accountable for too much, that you would possibly spotlighted for in the community. (Case D, Program Staff 1)*

Across the organizations there was a strong culture of mental health awareness and support. Staff challenged the stigma around mental health that many young people encountered in other workplaces and educational settings, with a focus on strengths-based approaches. In one of the WISEs there were specific tasks and workshops delivered on acceptance of differences and inclusivity, in all other cases these themes were observed in the operation and actions of staff. The message that young people encountered in all case studies is that *everyone faces different mental health, family, background challenges and this is a place where you can be yourself.* This creates a safe environment in which young people can feel supported to participate in group settings where different learning styles and ways of being are normalized. This level of acceptance was fostered throughout the organizations and was made explicit to new participants:

*I bring them up and I introduce them to [Name], [Name] what do you do? Right, and particularly the young ladies on [our training program] … their ears prick up. Because they can see this young lady doing all this magnificent high precision soldering and component replacement, and you watch them and you see their eyes stare … I say [Name], what were you doing five, six years ago, and she tells them, she calls herself an alcoholic, whether she was or wasn’t, she had trouble with alcohol and stuff like that. Fought with her mother, didn’t see her father, no job, no prospects, and that’s when the penny drops. (Case B, Manager 2)*

This authenticity was valued across the hierarchy of the case organizations. The senior staff were focused on providing a safe environment for young people where they could speak their mind and be open about any challenges they were facing. The most common approach employed was to ‘check in’ regularly:

*There’s been times here before where people will say, ‘[Name], are you okay today?’ Like [Leadership staff], last week, she said to me, ‘Are you okay today?’ I’m like, ‘Yeah, why’s that?’ And she’s like, ‘You’re not your happy, bubbly, like you want to be here - not saying you don’t want to be here, but are you sure you’re okay?’ … She definitely noticed [a difference]. And I’m like, ‘Yeah, I seem okay. I’m just a little – [Staff member] is leaving, and I’m just thinking a lot.’ She’s like, ‘Yeah, you’re just not your bright, bubbly’ - and I’m like, ‘Sorry. I don’t mean to be like that.’ And it really got me out of it. (Case D, Young person 1)*

The young people interviewed indicated they felt confident to express their mental health challenges and felt secure in the approach taken by staff. There was acknowledgement of the empathy being provided and the young people clearly felt a strong sense of connection and safety in the workplace. This level of collegiality and acceptance of the young people was something the young people really valued.

*It keeps you involved – to get involved with a community or to get involved at work, teamwork or in visually working. (Case C, Young person 8)*

*Yeah, just for them to have our backs all the time, I feel really supported. (Case A, Young person 3)*

Organisational culture was also reflected in organisational governance where on all boards there was a balance required between social goals and financial goals:

*‘I think the beauty and one of the reasons I really enjoy being involved in [the Board] is I feel like that balance is pretty well managed. The harmony between ensuring that we are not leaving any stone unturned around the profitability of the businesses but remembering why we’re there in the first place and ensuring the effectiveness of the social programs as well’.* (Director, Case A)

In none of the case WISEs were young people involved in governance processes given the transitional workplace model whereby most of the young people were not there as long-term employees. However, there was evidence of young people being given more authority and leadership roles in their organization, which enabled them to have some sort of influence among their peers, and for some a more direct supervisory role. This helped to build their self-efficacy and again contributed to an improved sense of well-being:

*In terms of the skills, I’ve definitely found that I’ve become more assertive… All the liaising that you need to do. Also leadership, not just of other people, but of yourself. Self-leadership is definitely a real thing. It’s all part of the motivation, the initiative. And on top of leadership, it’s teamwork skills and communication skills. (Case D, Young Person 1)*

*It helped me with a lot of things. It helped me with work. It helped me with sort of social stuff. It helped me de-stress. Work with different people that have never worked before, sort of being more – how do I say it – more of a role model. (Case D, Young person 6)*

The self-confidence and improvement in mental well-being through taking on a leadership and mentoring role was a common reflection of both staff and the young people interviewed. For continuing staff there was some opportunities provided for management type experience. Again, this was linked to an increased sense of confidence and self-esteem and reduced participants anxiety levels when confronted with new situations such as leadership opportunities.

### Policy and process

All the case study WISEs had in place processes and policies to support business operations and staff and participant wellbeing. Some of the key policies related to accommodating flexible work arrangements to suit employees and providing opportunities for learning through mistakes within certain expectation boundaries as captured in the following field notes:

*The rules of work apply… But if you cannot get transport to work there are alternatives to help you (youth support worker); if you conflict with someone there are people who can help you work this out (Trainers Assistant); if you do something wrong with the equipment you will be cautioned, but this will not be held against you (Trainer). In each of these cases the staff response secures the engagement of the student. This is the internal network that exists to support student participation in the program. (Researcher field notes)*

Providing flexible working conditions was one of the key processes identified in the organisations. This young person reflected on how they were offered the opportunity to take some time off after a ‘check in’ chat with a staff member:

*For example, on Monday, knowing that another staff member was leaving that week, and I was like, I don’t want them to go. I was a little up in my head too much. And I was like, great, it’s just like [previous organization name] all over again. And I just started getting a little bit of anxiety. And my manager said, ‘You’re really anxious today. I haven’t seen this in you since [previous organization name].’ And I’m like, ‘Yeah, it’s just everything happening at the moment.’ She’s like, ‘Do you need to go home?’ I’m like, ‘No, I’m fine.’ And I think being here, it really helps me. (Case D, Young person 1)*

Again, this relates to feeling safe within the workplace to share feelings and concerns and feeling validated to do so. For other young people a strong sense of structure was helpful for their mental health and well-being:

*Getting yourself in to a routine, can also help you get in to a routine of improving your physical health as well. (Case A, Young Person 6)*

*Before I came to this course I was like pretty depressed... Because I just kept getting knocked back, I was sort of like giving up. I definitely feel like healthier mentally just coming here every day and being punctual, having a routine. (Case B, Young person, 9)*

Another common reflection was the high degree of tolerance for making mistakes evident across all of the case WISEs which was a feature of the *Culture and Policies and Processes* of the organisations. Because of this, participants reported that they felt safe trying new approaches and expressing different ideas. This was particularly important for those participants who identified as neurodiverse, as inherent in their neurodiversity was that they saw the world differently and, thus, had different ideas and support requirements. Therefore, feeling safe to express their ideas and learn from mistakes was an important factor for them to feel a state of positive well-being and reduce their anxiety and depression:

*[The WISE] has been very lenient with my anxiety provoked mistakes. I do make pretty consistent mistakes. It’s good to have a sort of practice run. (Case A, Young person 2)*

A high degree of tolerance for mistakes also provided the young people with confidence that they could try and develop new skills. Some participants expressed that they felt valued for their contribution, despite their mental health challenges and their self-doubt. One participant expressed how staff were able to respond to changes in his mental health needs by providing support and encouragement and this impacted positively on his self-confidence:

*… The end of my first trial shift… [Name], who was supervising me at the time - I was worried I wasn’t doing too well, that I was kind of slower than everybody else and couldn’t get my bearings. He came and reassured me and he told me that everyone had actually told him that I was doing great. That was really good to hear and I walked out of there feeling pretty proud of myself. (Case A, Young Person 1)*

### Fostering local service networks

One of the important roles that the WISE managers and staff performed to support the young people was to develop relationships with local services and employers, and at times acting as the intermediating within those networks to help young people transition to open employment opportunities:

*‘I’ve got constant phone calls with [WISE Manager], so that’s been interesting and challenging and to be honest time consuming. But I think we’ve got a good working relationship . . . we can communicate and . . . tell each other . . . what’s working well, what’s not working so well’.* (Local employer and WISE partner organisation, Case D)

All of the WISEs performed some role in facilitating referrals to education, housing and welfare support providers on a case-by-case basis. Two of the case WISEs had a structured support program to help young people find work and in one WISE, they had staff attend the induction at the new workplace with young people which was a source of social and emotional support. Conversely, one of the case studies had spent considerable time and effort to establish industry relationships to support employment pathways but at this stage was struggling to see results for these efforts:

*Getting them through the Cert II is not the outcome. It's just an output and it's another pillar to help them move on and achieve further things … Therefore, what are the other support networks you need around it? … It's not something we can do on our own. We need commercial partnerships to be able to achieve it and it's getting that narration out there and that communication out there to get that support from commercial partners. That's when we'll really be able to really achieve what we're seeking. (Case D, Leadership)*

## Discussion

The perception of young people and staff were that their health and well-being had improved, particularly with respect to improved self-esteem and confidence, reduced anxiety and depression, and increased social connection. The purposeful design of the WISEs to create inclusive opportunities for work and training together with social networking opportunities is consistent with previous research [7, 52–54]. The findings presented in this paper extend this research by exploring the organisational features that enable these opportunities and health gains [10]. These include: deliberate organisational structures around staff support roles; provision of differing physical spaces to support mental health; a flexible and supportive organisational culture which is also reflected in the policies and processes (such as leave expectations for mental health); business design that enables a range of skills to be developed and an acceptance of learning from mistakes; and networking opportunities to assist employment transition. The findings suggest that the organisational design and space configuration influences health and well-being outcomes not merely having employment itself [55, 56].

One factor worth noting was the importance of a workplace that tolerates mistakes which made young people feel safe to try new activities and experiences, even if they were unsure about their ability level. This supports previous research that highlights the importance of tolerance for mistakes in developing psychological safety [57–59] and relates to perceived organizational support (POS) where employees feel that the organisation cares about their well-being, which is central to employee well-being [60, 61].

One of the main purposes of the organizations we studied seemed to be providing an inclusive workplace and training environment in order to engage young people from disadvantaged backgrounds where other organizations have failed to engage and retain them. This is different from other organizations, where an inclusive environment may be developed [62] but it is not the *reason* they exist. Inclusive practices were embedded in organizational features such as the structure, culture, policy, processes and practices. For instance, the case WISEs supported young people’s personal development via an organizational culture that was therapeutic, non-institutional, and that prioritized the ‘above and beyond’ provision of support; and through a structure that involved a dedicated youth programs team and wraparound support. This embedding and tweaking of ‘inclusivity’ – particularly towards tailored support – enabled the case WISEs to support the unique characteristics of youth participants as part of their core business model. Thus, there was evidence of both transactional processes whereby particular policies of the organisation such as flexible workplace arrangements were seen as supportive of health and well-being and more transformational processes, whereby the whole purpose and culture of the organisation was focused on providing an inclusive workplace environment [10].

A number of participants reflected that their organizations were providing a distinct workplace environment in relation to being inclusive, which was difficult to find elsewhere. Whether the organisations are having a transformational impact outside of their own organisation was less difficult to ascertain and apparently at this stage not being realised as much as staff would like. The difference in workplace environments was made explicit in one organization where they specifically created a transitions program with external host employers and discussed the ‘right kind’ of employment conditions for their young people. This was almost an explicit acknowledgement that the ‘right’ (inclusive, diverse, supportive) conditions do not exist in the ‘real world’ of work and they needed to find equally inclusive workplace environments for their young people to transition into. It is not apparent whether they were having much success in this regard.

### Strengths and limitations

The strength of this study was the use of an ethnographic approach to further understand in detail the organizational structures and processes to engage young people and positively impact their health and well-being as perceived by the young people themselves and the managers and staff [44]. One of the significant limitations of this study was the focus on a small number of cases, which raises the question of whether the findings would apply to other population groups and other groups of young people. Thus, future research could be undertaken to explore the extent to which certain organisational processes and structures lead to improved health and well-being outcomes for employees from disadvantaged backgrounds and with pre-existing mental health challenges. In addition, there was no quantifiable measure of health and well-being outcomes. The strength of this study was developing certain propositions on how these concepts relate [47] which could be tested with further research. Further research could also take a more participatory approach to the evaluation where young people themselves guide the research and evaluation questions whereas in this study the initial research questions were developed in a partnership approach with managers and support staff.

### Program and Policy Implications

One of the interesting potential applications concerning the data from this study relates to future considerations of replicating inclusive workplace designs. Clearly, there is a need for further research on the relationship between organisational design, structures, and processes and health and well-being outcomes. In designing this research, policy and practice considerations are paramount. What will be interesting to consider is *what* should be replicated. This relates to the concept of implementation fidelity, the degree to which real world implementation of the intervention adheres to the research conditions [63, 64]. In designing further research and then examining policy and practice implications, a key question is whether organisations should be assessed against the structures and processes seen in these case study organisations. There is an argument that for research and practice in complex environments the approach should be ‘*fidelity to function’* [65]. That is replicating the purpose or function of a strategy not its actual content [65]. In this case that would be thinking about health and well-being workplace objectives/goals when employing people from disadvantaged backgrounds and recognising that different workplaces would require different structures and processes to achieve these health and well-being goals dependent on the particular characteristics of the employees and industry focus. There maybe a set of core structures and processes for employing people from disadvantaged backgrounds and those previously excluded from mainstream employment and those that can be adapted for different contexts [64, 66]. Further appreciation of these systems concepts will have implications for research on organisational design and health and well-being outcomes.

## Conclusion

The findings of this study showed how perceived health and wellbeing gains were associated with certain organisational features such as a supportive organisational culture where young people were supported with mental health problems, a business design that enables a range of skills to be developed and an acceptance of learning from mistakes, different structures and spaces that enabled both socialising and solitude, and networking opportunities to assist employment transition. The findings highlight how the structure and processes of the organizations are related to health and well-being outcomes rather than just employment itself [10, 55]. Future research could explore some of the health and well-being outcomes that were uncovered in this study using quantitative methods and what organisational factors are important to replicate. Lastly, this study has revealed that hybrid/innovative business models, such as social enterprises, are an interesting area of research for inclusive workplaces and health and well-being outcomes and worthy of further investigation to expand on current theory and models.

## Data Availability

The datasets generated and analysed during the current study are not publicly available in raw form as part of the ethics requirements due to the small case sample size. Further information on the case studies are available at this link: https://www.csi.edu.au/research/project/improving-health-equity-young-people-role-social-enterprise/

## References

[1] Allen J, Balfour R, Bell R, Marmot M (2014). Social determinants of mental health. Int Rev Psychiatry.

[2] Gordon K, Wilson J, Tonner A, Shaw E (2018). How can social enterprises impact health and well-being?. Int J Entrep Behav Res.

[3] Morrow M, Wasik A, Cohen M, Perry KME (2009). Removing barriers to work: Building economic security for people with psychiatric disabilities. Crit Soc Policy.

[4] Green AE, Hasluck C (2009). Action to Reduce Worklessness: What Works?. Local Econ.

[5] Paluch T, Fossey E, Harvey C (2012). Social firms: Building cross-sectoral partnerships to create employment opportunity and supportive workplaces for people with mental illness. Work.

[6] Barraket J, Mason C, Blain B. Finding Australia’s Social Enterprise Sector 2016: Final Report. Melbourne, Australia: Social Traders and Centre for Social Impact Swinburne; 2016.

[7] Roy MJ, Lysaght R, Krupa TM (2017). Action on the social determinants of health through social enterprise. CMAJ Can Med Assoc J.

[8] Mason C, Roy MJ, Carey G (2019). Social enterprises in quasi-markets: exploring the critical knowledge gaps. Soc Enterp J.

[9] Roy MJ, McHugh N, Hill OC (2014). Social Innovation: Worklessness, Welfare and Well-being. Soc Policy Soc.

[10] Suchowerska R, Barraket J, Qian J, Mason C, Farmer J, Carey G, et al. An Organizational Approach to Understanding How Social Enterprises Address Health Inequities: A Scoping Review. J Soc Entrep. 2019:1–25.

[11] Shore LM, Randel AE, Chung BG, Dean MA, Holcombe Ehrhart K, Singh G (2011). Inclusion and Diversity in Work Groups: A Review and Model for Future Research. J Manag.

[12] Qian J, Riseley E, Barraket J (2019). Do employment-focused social enterprises provide a pathway out of disadvantage? An evidence review.

[13] Australia Institute of Health and Welfare. Young Australians: Their health and wellbeing 2011, Table of contents. 2011. https://www.aihw.gov.au/reports/children-youth/young-australians-their-health-and-wellbeing-2011/contents/table-of-contents.

[14] Solar O, Irwin A. A conceptual framework for action on the social determinants of health. 2010.

[15] Doroud N, Fossey E, Fortune T (2015). Recovery as an occupational journey: A scoping review exploring the links between occupational engagement and recovery for people with enduring mental health issues. Aust Occup Ther J.

[16] Hergenrather KC, Zeglin RJ, McGuire-Kuletz M, Rhodes SD (2015). Employment as a Social Determinant of Health: A Review of Longitudinal Studies Exploring the Relationship Between Employment Status and Mental Health. Rehabil Res Policy Educ.

[17] Waddell G, Burton AK. Is work good for your health and well-being? London, UK; 2006.

[18] Bharmal N, Derose KP, Felician M, Weden MM. Understanding the Upstream Social Determinants of Health. RAND Health. 2015; 20.

[19] Australian Institute of Health and Welfare. Australia’s health 2014. Australia’s health series no. 14. Cat. no. AUS 178. Canberra: AIHW; 2014.

[20] Elmes AI (2019). Health impacts of a WISE: a longitudinal study. Soc Enterp J.

[21] Ferguson KM, Xie B (2008). Feasibility study of the social enterprise intervention with homeless youth. Res Soc Work Pract.

[22] Ferguson KM (2018). Nonvocational Outcomes From a Randomized Controlled Trial of Two Employment Interventions for Homeless Youth. Res Soc Work Pract.

[23] Ferguson KM (2018). Employment Outcomes From a Randomized Controlled Trial of Two Employment Interventions With Homeless Youth. J Soc Soc Work Res.

[24] Agafonow A (2018). Setting the bar of social enterprise research high. Learning from medical science. Soc Sci Med.

[25] Roy MJ, Macaulay B, Donaldson C, Teasdale S, Baker R, Kerr S (2018). Two false positives do not make a right: Setting the bar of social enterprise research even higher through avoiding the straw man fallacy. Soc Sci Med.

[26] Chandra Y (2018). Social enterprise for the visually impaired: Voices from within. Int J Disabil Hum Dev.

[27] Macaulay B, Mazzei M, Roy MJ, Teasdale S, Donaldson C (2018). Differentiating the effect of social enterprise activities on health. Soc Sci Med.

[28] Barraket J, Douglas H, Eversole R, Mason C, McNeill J, Morgan B (2017). Classifying social enterprise models in Australia. Soc Enterp J.

[29] Barraket J, Moussa B, Campbell P, Suchowerska R. How Do Social Enterprises Influence Health Equities? A Comparative Case Analysis. In: Social Enterprise, Health, and Wellbeing. Routledge; 2021.

[30] Joyce A, Elmes A, Campbell P, Moussa B, Suchowerska R, Barraket J (2022). The health and well-being impacts of a work integration social enterprise from a systems perspective. Health Promot Int.

[31] Lysaght R, Roy MJ, Rendall JS, Krupa T, Ball L, Davis J (2018). Unpacking the foundational dimensions of work integration social enterprise The development of an assessment tool. Soc Enterp J.

[32] Janssen S, Tahitu J, van Vuuren M, de Jong MDT (2018). Coworkers’ Perspectives on Mentoring Relationships. Group Organ Manag.

[33] Levitt H, Bamberg M, Creswell J, Frost D, Josselson R, Suárez-Orozco C (2018). Journal Article Reporting Standards for Qualitative Primary, Qualitative Meta-Analytic, and Mixed Methods Research in Psychology: The APA Publications and Communications Board Task Force Report. Am Psychol.

[34] Liu G, Rong K (2015). The Nature of the Co-Evolutionary Process: Complex Product Development in the Mobile Computing Industry’s Business Ecosystem. Group Organ Manag.

[35] Australian Government Department of Health (2019). National Action Plan for the Health of Children and Young People: 2020–2030.

[36] Australian Bureau of Statistics. Main Features - Labour Force Commentary July 2020. 2020. https://www.abs.gov.au/ausstats/abs@.nsf/7d12b0f6763c78caca257061001cc588/a8e6e58c3550090eca2582ce00152250!OpenDocument. Accessed 27 Aug 2020.

[37] Stake R, Denzin NK, Lincoln Y (2008). Qualitative case studies. Strategies of qualitative inquiry.

[38] Yin RK (2013). Case Study Research: Design and Methods.

[39] Flyvbjerg B (2006). Five Misunderstandings About Case-Study Research. Qual Inq..

[40] Australian Bureau of Statistics. 4430.0 - Disability, Ageing and Carers, Australia: Summary of Findings, 2015. Canberra, Australia; 2016.

[41] Geertz C (1973). The Interpretation of Cultures.

[42] Cook KE (2005). Using critical ethnography to explore issues in health promotion. Qual Health Res.

[43] Tracey P, Phillips N, Jarvis O (2011). Bridging Institutional Entrepreneurship and the Creation of New Organizational Forms: A Multilevel Model. Organ Sci.

[44] Gioia DA, Corley KG, Hamilton AL (2013). Seeking Qualitative Rigor in Inductive Research: Notes on the Gioia Methodology. Organ Res Methods.

[45] Bryman A (2004). Social Research Methods.

[46] Gobo G. Sampling, Representativeness and Generalizability. In: Qualitative Research Practice. 1 Oliver’s Yard, 55 City Road, London England EC1Y 1SP United Kingdom: SAGE Publications Ltd; 2004. p. 405–26.

[47] Gioia DA, Chittipeddi K (1991). Sensemaking and Sensegiving in Strategic Change Initiation. Strateg Manag J.

[48] Patton M (2002). Qualitative Research & Evaluation Methods.

[49] Nutbeam D, Bauman A (2006). Evaluation in a nutshell.

[50] Corbin JM, Strauss A (1990). Grounded theory research: Procedures, canons, and evaluative criteria. Qual Sociol.

[51] Blaikie N. Designing social research : the logic of anticipation. 2nd edition. Cambridge, UK: Malden, Mass: Polity; 2009.

[52] Butterworth P, Leach LS, Strazdins L, Olesen SC, Rodgers B, Broom DH (2011). The psychosocial quality of work determines whether employment has benefits for mental health: results from a longitudinal national household panel survey. Occup Environ Med.

[53] Chandler J. A study to explore the impact of working in a social enterprise on employee health and wellbeing in Greater Manchester. PhD. University of Salford; 2016.

[54] Hazenberg R, Seddon F, Denny S (2014). Investigating the Outcome Performance of Work-Integration Social Enterprises (WISEs): Do WISEs offer “added value” to NEETs?. Public Manag Rev.

[55] Farmer J, De Cotta T, McKinnon K, Barraket J, Munoz S-A, Douglas H (2016). Social enterprise and wellbeing in community life. Soc Enterp J.

[56] Farmer J, De Cotta T, Kamstra P, Brennan-Horley C, Munoz SA (2019). Integration and segregation for social enterprise employees: A relational micro-geography. Area.

[57] Edmondson A (1999). Psychological Safety and Learning Behavior in Work Teams. Adm Sci Q.

[58] Weinzimmer LG, Esken CA (2017). Learning From Mistakes: How Mistake Tolerance Positively Affects Organizational Learning and Performance. J Appl Behav Sci.

[59] Zeng H, Zhao L, Zhao Y (2020). Inclusive Leadership and Taking-Charge Behavior: Roles of Psychological Safety and Thriving at Work. Front Psychol.

[60] Kurtessis JN, Eisenberger R, Ford MT, Buffardi LC, Stewart KA, Adis CS (2017). Perceived Organizational Support: A Meta-Analytic Evaluation of Organizational Support Theory. J Manag.

[61] Neves P, Eisenberger R (2014). Perceived organizational support and risk taking. J Manag Psychol.

[62] Shore LM, Cleveland JN, Sanchez D (2018). Inclusive workplaces: a review and model. Hum Resour Manag Rev.

[63] Morrel-Samuels S, Rupp LA, Eisman AB, Miller AL, Stoddard SA, Franzen SP (2018). Measuring the Implementation of Youth Empowerment Solutions. Health Promot Pract.

[64] van Daele T, van Audenhove C, Hermans D, van den Bergh O, van den Broucke S (2014). Empowerment implementation: enhancing fidelity and adaptation in a psycho-educational intervention. Health Promot Int.

[65] Hawe P (2015). Lessons from Complex Interventions to Improve Health. Annu Rev Public Health.

[66] Hoekstra F, van Offenbeek MAG, Dekker R, Hettinga FJ, Hoekstra T, van der Woude LHV (2017). Implementation fidelity trajectories of a health promotion program in multidisciplinary settings: managing tensions in rehabilitation care. Implement Sci.

